# Long-Term Health-Related Quality of Life Outcomes Following Thyroid Surgery for Malignant or Benign Disease: Deficits Persist in Cancer Survivors Beyond Five Years

**DOI:** 10.1007/s00268-022-06643-5

**Published:** 2022-07-07

**Authors:** Nicholas D. A. Blefari, Christopher W. Rowe, Elvina Wiadji, David Lambkin, Rosemary Carroll, Elizabeth A. Fradgley, Christine J. O’Neill

**Affiliations:** 1 Surgical Services, John Hunter Hospital, Locked Bag 1, Hunter Regional Mail Centre, Newcastle, NSW 2310 Australia; 2 Department of Endocrinology, John Hunter Hospital, Newcastle, NSW Australia; 3 University of Newcastle, Newcastle, NSW Australia; 4 Hunter Medical Research Institute, Newcastle, NSW Australia

## Abstract

**Background:**

Thyroid cancer diagnoses are increasing and treatment can lead to significant morbidity. Long-term health-related quality of life (HRQoL) in thyroid cancer is understudied and lacks reference populations. This study compares long-term HRQoL between patients with thyroid cancer or benign disease, following thyroid surgery.

**Methods:**

Patients undergoing thyroidectomy between 2000 and 2017 were identified from a pathology database. 696 participants (278 malignant, 418 benign) were invited to complete a validated disease-specific HRQoL tool, City of Hope—Thyroid Version. Propensity scores were used to adjust for demographic and clinical differences between cohorts.

**Results:**

206 patients (102 malignant, 104 benign), 71% female, returned surveys a median of 6.5 (range 1–19) years after thyroidectomy. Of the cancer cohort, 95% had differentiated thyroid cancer and 83% remained disease-free. There were no significant differences in overall HRQoL scores between groups. In comparison to the benign cohort, cancer patients showed a significant detriment in the social subdomain score (OR 0.10–0.96, *p* = 0.017) but not in other subdomains (physical, psychological, spiritual). Female gender, increasing BMI and cancer recurrence were significantly associated with decreased overall HRQoL. Compared to the benign cohort, cancer patients reported more personal and family distress associated with diagnosis and treatment, increased future uncertainty, poorer concentration and greater financial burden.

**Conclusion:**

Although no difference in overall HRQoL was found between patients undergoing thyroidectomy for benign or malignant disease, detriments in social well-being may persist many years after surgery. Thyroid cancer patients and their families may benefit from increased supports around the time of diagnosis and treatment.

**Supplementary Information:**

The online version contains supplementary material available at 10.1007/s00268-022-06643-5.

## Introduction

The incidence of thyroid cancer has increased substantially over the last few decades, largely driven by an increase in incidental diagnoses of papillary thyroid carcinoma (PTC) [1–3]. For the majority of patients, thyroid cancer is associated with excellent survival (> 97% ten-year survival for those with low-risk disease) [4, 5]. In the past, all patients with thyroid cancer were treated with total thyroidectomy and radioiodine ablation (RAI); however, recent evidence has suggested that for those with low-risk differentiated thyroid cancer (DTC), thyroid lobectomy confers similar overall survival rates and only a small increase in risk of recurrence [6–9]. The rationale behind this de-escalation of care has not only been improved knowledge about natural history of the disease, but also an appreciation of treatment related side effects and health-related quality of life (HRQoL) detriments that can occur in patients with thyroid cancer [10, 11]. Furthermore, thyroid cancer patients have been found to have equivalent if not worse HRQoL detriments when compared to breast or colon cancer survivors, who have an inferior prognosis [12–14].

Multiple studies have examined HRQoL outcomes in thyroid cancer survivors [15–19]. Issues with current studies include small cohorts, retrospective identification of patients, use of generic rather than thyroid-specific HRQoL instruments, and the lack of a control group [20, 21]. Most studies have short-term follow up (< 5 years), yet the negative effects of a thyroid cancer diagnosis can continue for many years [16, 22, 23]. Whilst there have been studies comparing HRQoL in thyroid cancer patients to survivors of other malignancies [12–14], there is a lack of data comparing thyroid cancer survivors to those undergoing surgery for benign thyroid disease. Despite potential differences in pre-operative compressive or thyrotoxic symptomatology, patients with benign thyroid disease represent a control comparator for the intervention of total thyroidectomy, but without the psychosocial implications and follow-up requirements associated with a cancer diagnosis.

This study is the first to quantitatively assess HRQoL outcomes in an Australian cohort of thyroid cancer patients, selected by their pathology and geography only. We have also compared the HRQoL of thyroid cancer patients to a cohort of patients who underwent surgery for benign disease. In addition to documenting HRQoL in these groups, we aimed to identify clinical or demographic factors that increase the likelihood of HRQoL detriments.

## Methods

**Fig. 1:**
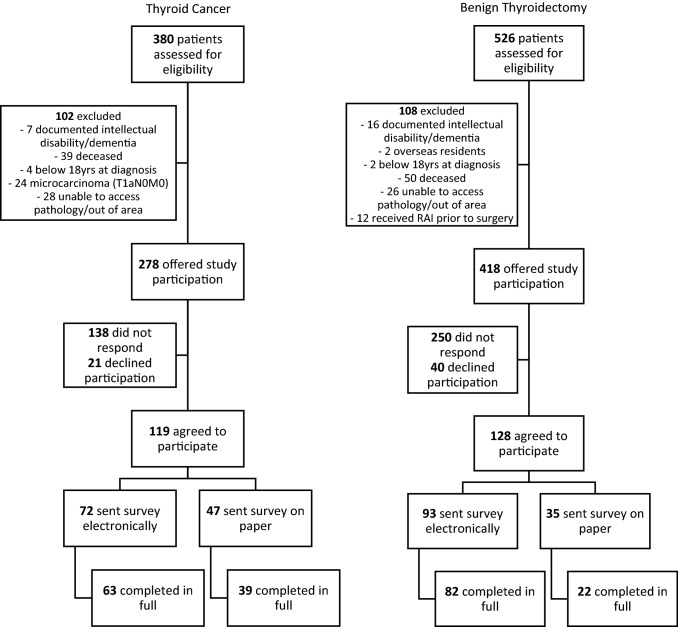
Study flow

Patients who underwent surgical treatment for primary thyroid cancer or total thyroidectomy for benign thyroid disease between 2000 and 2017 within a single health district in New South Wales (NSW), Australia, were identified from a histopathology database (NSW Health Pathology—Hunter, NSW Health). The health district (Hunter New England Local Health District) has an area of 131,785 km^2^ with an estimated population of 920,000, spanning metropolitan, regional and rural areas. Potential participants were screened for eligibility using electronic medical record databases and thyroid cancer multidisciplinary meeting records. Excluded patients were aged < 18 years at the time of surgery, deceased or cognitively impaired. Patients with a diagnosis of papillary thyroid microcarcinoma (PTMC) limited to the thyroid were also excluded as local clinical practice is of a restrictive biopsy protocol that avoids biopsy of nodules < 1 cm, and follow-up is not offered to the majority of patients with incidental PTMC. Patients who under total thyroidectomy for benign disease (confirmed on histopathology) were recruited as a control group. These were selected to control for the intervention ‘total thyroidectomy’, which was the extent of surgery recommended at the time for patients with papillary thyroid cancer > 1 cm [24]. A total of 696 (278 cancer and 418 benign) eligible patients were mailed an invitation to participate in the study. Completed consent forms were returned via mail to the study team, and consenting patients were sent the study questionnaire electronically or on paper, depending on participant preferences (Fig. 1). No reminders were sent and data collection in both groups was completed prior to the COVID-19 pandemic.

Participants were asked to complete a validated thyroid-specific questionnaire, the City of Hope-Thyroid Version [25]. This survey has been used in prior HRQoL studies [22, 26], and is one of only two available thyroid cancer specific tools that have undergone Phase IV testing [21]. It provides an overall well-being averaged score, along with averaged sub-scores in four domains (physical symptoms, psychological well-being, social concerns, and spiritual well-being). Scores are presented using a Likert scale from 0 to 10 with anchors such as ‘no problem’ or ‘severe problem’ provided; reverse anchors are included but are flipped in analysis so that higher HRQoL scores represent better outcomes. Within the psychological well-being domain there were 8 cancer-specific items that were only asked of patients in the malignant group (distress associated with RAI ablation, whole body scanning, thyroglobulin testing or thyroid hormone withdrawal; or fear of future diagnostic tests, a second cancer, recurrence or metastasis). Sensitivity analyses were performed comparing the overall and psychological mean scores excluding these 8 questions between groups, to ensure the inclusion of these items for the malignant group only did not influence the mean scores of the primary analyses. All items in the physical, social and spiritual domains were answered by all participants, regardless of a benign or malignant diagnosis.

Additional survey items captured patient demographics (level of education, BMI, smoking status), current doses of thyroxine, treatment complications, survey completion time and satisfaction levels. Clinical (including details of surgery, RAI and treatment complications) and pathological data was collected from the electronic medical record. Study data were collected using Research Electronic Data Capture (REDCap®) database [27]. The study and its protocol were prospectively approved by the Hunter New England Human Research Ethics Committee (2018/ETH00470) and University of Newcastle Human Research Ethics Committee (H2021-0059). All participants provided written informed consent.

Descriptive statistics are presented, comparing HRQoL scores for both malignant and benign cohorts, both overall and stratified by demographic and disease-specific data. Propensity scores were used to adjust for differences between demographic and clinical characteristics in the cancer and benign cohorts. Prespecified characteristics included in the model were age, BMI, gender, indigenous status, smoking status, education, time since diagnosis and most recent thyroid stimulating hormone (TSH) result. Unadjusted and adjusted scores were compared using least square means. An alpha level of 0.05 was specified for statistical significance. Data were analysed using SAS v9.4. (SAS Institute, Cary, North Carolina, USA).

## Results

### Survey response rates and participant demographics

Survey responses were received from 102 (37%) of potentially eligible patients with thyroid cancer and 104 (25%) of potentially eligible patients in the benign surgical cohort (Fig. 1). Survey responders had similar basic demographics to survey non-responders but were slightly older (51.4 vs 49.1yrs, *p* = 0.04, Supplementary Table 1). Within the study group, patients with thyroid cancer were more likely to be male (37% vs 20%, *p* = 0.007) and of a lower BMI (29.3 vs 31.5, *p* = 0.02) than those with benign disease, Table 1. Patients with benign disease completed the survey an average of 8.2 years (vs 6.8 years for patients with cancer, *p* = 0.04) after their surgical date and were more likely to complete the survey electronically rather than on paper, (79% vs 62%, *p* = 0.007, Table 1). There was no difference in most recent TSH between cohorts (1.56 vs. 1.55, *p* = 0.98, Table 1). Patients in the thyroid cancer cohort were more likely to have a suppressed TSH (less than 0.3mIU/L) at the time of survey completion, but this was not statistically significant (20 (25%) vs. 11 (14%), *p* = 0.09). Participant indications for surgery and disease characteristics are summarised in Table 2. Interestingly, 2 patients in the benign group reported that they had thyroid cancer, although this did not correlate with their operative histopathology or electronic medical record.

**Table 1:** Participant demographics

Characteristic	Thyroid cancer [*n* = 102]	Benign thyroidectomy [*n* = 104]	Total [*n* = 206]	*p* value
Age (years) at survey completion (mean, SD)	60.9 (14.1)	59.9 (13.8)	60.4 (13.9)	0.59
Sex (female, %)	64 (63%)	83 (80%)	147 (71%)	0.007*
Indigenous (*n*, %)	4 (3.9%)	3 (2.9%)	7 (3.4%)	0.68
BMI (kg/m^2^) (mean, SD)	29.3 (6.9)	31.5 (6.9)	30.4 (6.9)	0.02*
Time (years) since diagnosis (mean, SD)	6.8 (4.6)	8.2 (5.2)	7.5 (4.9)	0.04*
Most recent TSH (normal range 0.4–4.0mIU/L) (mean, SD)	1.56 (1.78) [*n* = 80]	1.55 (2.04) [*n* = 77]	1.55 (1.91)	0.98
Current smoker (*n*, %)	6 (6%)	7 (6.8%)	13 (6.4%)	0.20
Highest level of education achieved (*n*, %)	*n* = 100	*n* = 103	*n* = 203	
*Did not complete high school*	17 (17%)	16 (16%)	33 (16%)	0.47
*Completed high school*	26 (26%)	36 (35%)	62 (31%)	
*Diploma/TAFE*^b^ *certificate*	32 (32%)	34 (33%)	66 (33%)	
*Undergraduate/bachelor degree*	17 (17%)	13 (13%)	30 (15%)	
*Postgraduate degree* *(masters, PhD *etc*.)*	8 (8%)	4 (4%)	12 (6%)	
Survey completion method–electronic (n, %)	63 (62%)	82 (79%)	145 (70%)	0.007*
Self-reported survey completion time–mins (mean, SD)	16 (10)	16 (15)	16 (13)	0.95
Survey satisfaction score^a^ (mean, SD)	7.1 (2.5)	7.0 (2.5)	7.0 (2.5)	0.77

^a^Survey satisfaction was reported on a Likert scale, with 0 being the worst possible and 10 being the best possible result

^b^*TAFE* Technical and further education (Australia) refers to vocational training

**Table 2:** Disease characteristics

Characteristic	Thyroid cancer [*n* = 102]	Benign thyroid disease [*n* = 104]
*Procedure type (n, %)*		
Total thyroidectomy	71 (70%)	104 (100%)
Hemithyroidectomy + completion thyroidectomy Hemithyroidectomy alone	27 (26%) 4 (4%)	– –
Tumour size in mm (mean, SD)	28.8 (17.7)	–
*Tumour type (n, %)*		
Papillary thyroid carcinoma	71 (70%)	–
Follicular thyroid carcinoma	19 (19%)	–
Hurthle cell carcinoma	7 (7%)	–
Medullary thyroid carcinoma	5 (6%)	–
*UICC/AJCC TNM stage (8th ed.)*		
Stage I	69 (68%)	–
Stage II	26 (25%)	–
Stage III	3 (2.9%)	–
Stage IV	4 (4.0%)	–
Thyroid cancer structural recurrence during follow–up period	17 (17%)	–
*ATA risk score stratification (n, %)*		
Low	69 (68%)	–
Intermediate	16 (16%)	–
High	12 (12%)	–
Not applicable^a^	5 (5%)	–
*Received radioactive iodine, RAI (n, %)*	84 (82%)	–
Cumulative total RAI dose (mCi) (median, range) [*n* = 76]	3850 (1000, 24,800)	–
^b^Single low dose, GBq (mean, SD) [*n* = 7]	1071 (49)	–
^b^Single high dose, GBq (mean, SD) [*n* = 54]	3633 (594)	–
Multiple doses, GBq (mean, SD) [*n* = 15]	11 687 (5985)	–
*Operative indication—benign thyroid disease (n, %)*		
Toxic multinodular goitre	–	16 (15%)
Goitre, non–toxic	–	57 (54%)
Graves’ disease	–	23 (22%)
Thyroiditis	–	8 (7.7%)

^a^Medullary thyroid carcinoma

^b^Low dose RAI defined as < 1500 GBq; High dose > 1500 GBq

### HRQoL scores

The mean overall HRQoL score for participants in the study population was 6.5 (on a scale of 0 to 10 with higher scores reflecting better HRQoL), with subdomain scores of 7.0 (physical), 6.8 (psychological), 7.8 (social) and 4.3 (spiritual). For the overall HRQoL score, there was no difference in unadjusted or adjusted scores between the thyroid cancer and benign thyroid disease cohorts (Table 3). However, after multivariate propensity score adjustment, there was a significant difference in patient-reported HRQoL within the social domain, with the benign cohort reporting better HRQoL scores, 8.1 (7.8–8.5) than cancer patients, 7.6 (7.2–7.9; *p* = 0.017).

**Table 3:** HRQoL Scores (adjusted and unadjusted) plus estimate of size of effect (Cohen’s D)

	Thyroid cancer Estimate (95% CI)	Benign thyroid Estimate (95% CI)	*p* value	Cohen’s D
*Overall QoL Score*				
Unadjusted	6.46 (6.21–6.70)	6.50 (6.26–6.75)	0.79	0.037
Adjusted	6.45 (6.20–6.70)	6.60 (6.36–6.85)	0.39	0.12
*Physical Well-Being QoL Score*				
Unadjusted	7.11 (6.74–7.48)	6.84 (6.47–7.20)	0.30	0.15
Adjusted	7.16 (6.80–7.53)	6.91 (6.55–7.28)	0.34	0.13
*Psychological Well-Being QoL Score*				
Unadjusted	6.63 (6.31–6.96)	6.91 (6.59–7.23)	0.23	0.17
Adjusted	6.62 (6.29–6.94)	7.02 (6.70–7.34)	0.083	0.24
*Social Well-Being QoL Score*				
Unadjusted	7.58 (7.25–7.90)	7.98 (7.66–8.31)	0.085	0.24
Adjusted	7.60 (7.29–7.92)	8.14 (7.83–8.45)	0.017*	0.33
*Spiritual Well-Being QoL Score*				
Unadjusted	4.45 (4.09–4.81)	4.19 (3.83–4.54)	0.31	0.14
Adjusted	4.36 (3.99–4.73)	4.26 (3.90–4.63)	0.72	0.05

Model was adjusted for age, BMI, sex, Indigenous status, smoking status, education, time since diagnosis and most recent TSH result

In addition, an exploratory analysis of specific questions identified significant differences between the cancer cohort and benign cohort (individual items with significant differences reported in Table 4, full list of items reported in Supplementary Table 2). Interestingly, thyroid cancer survivors rated their overall physical health as better than those with benign thyroid disease (6.5 (2.0) vs 5.7 (1.9), *p* = 0.005). On most other individual items listed in Table 4, cancer survivors reported lower scores than those with benign disease. These questions included items related to distress around the time of diagnosis and treatment, the ability to concentrate, fear of the future and impacts on family and finances.

**Table 4:** Exploratory analysis of individual HRQoL items of clinical significance

	Thyroid cancer Mean (SD)	Benign thyroid Mean (SD)	*p* value
Rate your overall physical health	6.5 (2)	5.7 (1.9)	0.005^**a**^
How is your present ability to concentrate or remember things?	6.1 (2.5)	5.4 (2.4)	0.03
How distressing were the following aspects of your treatment? *Initial diagnosis* *Surgery* *Time since treatment was completed*	3.8 (3.2) 4.4 (2.9) 7 (2.7)	5.8 (3.4) 5.5 (3.3) 8.1 (2.3)	** < **0.0001 0.02 0.006
How fearful are you of future diagnostic tests?	7.4 (2.7)	8.5 (2.4)	0.003
How distressing has your thyroid illness been for your family?	5.9 (3.1)	7.6 (2.9)	0.0001
How much isolation has your illness/treatment caused?	8.2 (2.9)	9 (2.1)	0.04
How much financial burden have you incurred as a result of your illness/treatment?	7.1 (3.3)	8 (2.7)	0.04
How important to you is your participation in religious activities?	2.8 (3.8)	1.7 (3.1)	0.03^**a**^
How much has your spiritual life changes as a result of thyroid disease diagnosis?	3.8 (3.1)	2.9 (3.5)	0.03^**a**^
How much uncertainty do you feel about your future?	6.9 (3)	8.4 (2.5)	0.0002

^**a**^These 3 items reported higher scores in the cancer group compared to the benign group. All other items are significant for lower scores in the cancer group

### Hypoparathyroidism

There were 6 patients in this study [3 malignant, 3 benign] who self-reported permanent hypoparathyroidism (defined as need for calcium and/or calcitriol supplements more than 6 months after surgery). Overall HRQoL scores were lower in the hypoparathyroid group, mean 5 (1.7) versus 6.5 (1.3). Across all subdomain scores, self-reported hypoparathyroidism was associated with lower HRQoL [physical 6 (2.9) vs 7 (1.9); psychological 4.9 (1.5) vs 6.8 (1.7); social 5.7 (1.8) vs 7.8 (1.7); and spiritual 3.3 (1.9) vs 4.3 (1.8)].

### HRQoL in the cancer group

When clinical and demographic variables were compared to HRQoL in the cancer group, female gender, increased BMI and shorter time interval between surgery and time of survey were associated with a statistically significant worse overall HRQoL, (*p* = 0.02, 0.02 and *p* = 0.0003, respectively, Table 5). Patient age and extent of surgery were not associated with HRQoL. There was a trend between increasing stage of disease and inferior HRQoL (*p* = 0.05 unadjusted, 0.06 adjusted). Cancer recurrence and was strongly associated with decreased HRQoL in this cohort (*p* = 0.0003).

**Table 5:** Associations between demographic, clinical characteristics and overall quality of life scores (thyroid cancer group only, *n* = 102)

Parameter (comparison)	Number	Unadjusted estimate (95% CI)	*p* value	Adjusted estimate (95% CI)	*p* value
Age at survey completion (per 1 year increase)	102	0.01 (− 0.01, 0.03)	0.35	0.00 (− 0.01 to 0.02)	0.63
Sex (male vs. female)	102	0.30 (− 0.24, 0.84)	0.27	0.57 (0.07–1.07)	**0.024**^a^
Time since surgery (per 1 year increase)	102	0.02 (− 0.03, 0.08)	0.39	0.07 (0.01–0.13)	**0.023**^a^
TNM 8th Ed stage (Stage III/IV vs stage I)	102	− 1.04 (− 2.07, − 0.01)	**0.048**	− 1.03 (12.12, − 0.06)	0.063
Extent of surgery (Hemi vs total)	102	0.27 (− 0.31, 0.85)	0.36	− 0.09 (− 0.65 to 0.46)	0.73
Recurrence of cancer (Yes vs No)	102	− 1.10 (− 1.77, − 0.43)	**0.0013**	− 1.61 (− 2.48, − 0.73)	**0.0003**^a^
RAI treatment (single high dose vs. no RAI or single low dose)	94	0.02 (− 0.62, 0.66)	0.95	− 0.32 (− 0.91, 0.26)	0.28
Level of education (did not complete high school vs. HSC or equivalent)	100	0.17 (− 0.63, 0.97)	0.68	0.04 (− 0.67, 0.74)	0.92
BMI (per 1 unit increase)	95	− 0.07 (− 0.11, − 0.03)	**0.0002**	− 0.06 (− 0.10, − 0.03)	**0.0003**^a^
Smoking status (current or stopped < 10 years ago vs. never smoked or stopped > 10 years ago)	100	− 0.40 (− 1.12, 0.33)	0.28	− 0.34 (− 1.01, 0.33)	0.32

Bold indicates statistically significant p value (p=<0.05)

^a^Decreased HRQoL significantly associated with female gender, survey time closer to time of surgery, cancer recurrence and increasing BMI

## Discussion

With an increasing number of thyroid cancer survivors, it is imperative that the long-term ramifications of treatment for thyroid cancer are well described and taken into account from the time of diagnosis [10, 11]. This study adds to the growing body of literature reporting long-term HRQoL in such patients and is the one of the first studies to compare HRQoL between patients who have been treated for primary thyroid malignancy to those who underwent surgery for benign thyroid disease. Due to the nature of recruitment for this study, where patients underwent surgery between 2000 and 2017, most cancer patients underwent total thyroidectomy and received radioactive iodine. This was consistent with international guidelines at the time [24]. Because of this we selected a control group for the intervention ‘total thyroidectomy’ amongst patients with benign disease. Despite finding no difference in overall HRQoL scores, in this study, cancer patients were shown to have persisting HRQoL deficits relating to social functioning, many years after their surgery.

To our knowledge, this study is only the second published that compares HRQoL in patients with benign and malignant thyroid disease. Giusti et al. [28] used the ThyPRO tool [29] to evaluate HRQoL yearly for 5 years in 123 thyroid cancer survivors, and compared these to 192 controls who had undergone thyroid surgery for benign disease. Similar to our study, no overall difference in HRQoL was found between the cohorts; and an association between a higher BMI and lower HRQoL was seen. However, as ThyPRO is a tool that was designed for use in patients with benign disease, issues pertinent to a cancer diagnosis and disease recurrence were not assessed.

The City of Hope QoL tool, as used in our study, has previously been used in The North American Thyroid Cancer Survivorship Study (NATCSS), reporting data on 1174 North American thyroid cancer survivors recruited from clinics, survivorship groups and social media [22]. Overall and subdomain HRQoL scores in our Australian cohort of thyroid cancer survivors were higher when compared to NATCSS, measuring 6.5 versus 5.6 overall, and subdomain scores of 7.1 versus 5.8 (physical), 6.6 versus 5.0 (psychological) and 7.6 versus 6.5 (social). Only the spiritual subdomain score in our study was lower than NATCSS (4.3 vs 5.6). Some of these differences may be explained by differences in sociodemographic factors, recruitment strategies and the shorter time-interval from surgery in NATCSS (51% of participants 3 years or less); yet it remains of interest that HRQoL scores were consistently higher in Australian patients in all subdomains with the exception of the spiritual domain. Cultural differences may contribute to the low scores in the spiritual domain and may affect the future utility of this subdomain in locations outside of North America. Other similarities between these two studies include low self-reported HRQoL scores in variables such as fear associated with surgery, fear of cancer recurrence and impact on family.

Fear of cancer recurrence and lack of social supports have previously been identified as significant drivers of poor HRQoL in thyroid cancer survivors [17, 30–32]. Papaleontiou et al. [33] reported high rates of ‘worry’ about death, cancer recurrence, treatment related side-effects, and family impacts amongst disease-free thyroid cancer survivors. In this current study, the most significant HRQoL detriments in cancer survivors related to distress around the time of diagnosis and surgery, fear of future tests and future uncertainty as well as the effect of the disease on their family. These findings are extremely important, as they represent many of the ‘key encounters’ that patients with a diagnosis of cancer would experience throughout their treatment journey. They also represent key points in time where supports could be offered. Supportive care with psychosocial interventions is an unmet need in thyroid cancer survivors. Despite extensive research in other cancer subtypes focussing on interventions (such as different forms of counselling like cognitive behavioural therapy) aimed at improving physical, psychological and social HRQoL [34, 35], there is only emerging evidence of use or effectiveness of psychosocial interventions in thyroid cancer [36].

This study has several limitations. The survey completion rate is lower than many in the published literature and was likely driven by length of time from treatment and the recruitment methods used. Recruitment was limited by information available on digital medical records (accuracy of contact details could not be checked), and initial non-responders were not followed after initial lack of response. However, comparison of gender, time since surgery and age at diagnosis, demonstrate that the participants are likely to be a demographically-representative sample of the eligible cohort. Comparison of other factors that may affect the representativeness of our sample cohort (such as patient satisfaction or pre-operative symptomatology) could not be evaluated. Most of the patients with thyroid cancer and all of those in the benign group underwent total thyroidectomy, thus HRQoL differences between total thyroidectomy and thyroid lobectomy cannot be accurately evaluated in this study. Participants in this study were surveyed only at a single time point, often many years after their surgery; there are no baseline results for comparison. There was also variation between survey completion and time since treatment. Whilst this limits comparison between participants at any set time point, it does allow time since treatment to be analysed as a variable against HRQoL results. We acknowledge that there may be response shift associated with individuals normalising the health deficits associated with their thyroid condition over time.

The City of Hope—Thyroid Version [25] was not specifically designed for patients with benign thyroid disease. However, the research team felt that despite this, the tool contains questions which are thyroid-specific and not necessarily cancer-specific, and thus that this tool would comprehensively compare these two groups. In addition, as only a disease-specific tool has been used, results cannot be generalised to population groups or other cancer survivors. We chose to focus on a comparative research question between two groups, as opposed to a descriptive question within a single patient group. This decision was driven by the wide-spread perception that thyroid cancer is a ‘good cancer’ with low mortality rates, and survivors may not experience worse outcomes than those with other thyroid conditions. In order to confirm or challenge this perception, which has implications for patient communication and follow-up, it is important to have an appropriate comparison group.

Despite these limitations, this study provides long-term, real-life data on HRQoL in a cohort of thyroid cancer patients selected by their geography, rather than being within a single academic centre or recruited via survivorship groups. The comparison to a control group for treatment effects (surgery) provides unique and novel insights into HRQoL detriments that will direct further research.

## Conclusion

The majority of thyroid cancer patients can expect long-term survival. It is imperative that they are given full opportunity to regain long-term HRQoL across all domains. Whilst this study shows that overall, HRQoL is preserved for the majority, clinicians should be aware of the significant social impacts that surround a diagnosis of thyroid cancer. It is particularly noteworthy that fear of cancer recurrence remains a significant issue for patients many years after diagnosis. Further research should focus on interventions that provide targeted supports to thyroid cancer survivors.

## Supplementary Information

Below is the link to the electronic supplementary material.

Supplementary file1 (DOCX 17 kb)
