# Supplementary material for: Long-Term Health-Related Quality of Life Outcomes Following Thyroid Surgery for Malignant or Benign Disease: Deficits Persist in Cancer Survivors Beyond Five Years
Source: World J Surg. 2022 Jul 7;46(10):2423–32. doi: 10.1007/s00268-022-06643-5 (PMC9261884; doi:10.1007/s00268-022-06643-5)
Supplement: Supplementary file 1 — Supplementary file1 (DOCX 17 kb) [file 268_2022_6643_MOESM1_ESM.docx]

**Supplementary Table 1: Comparison of demographics between study participants and eligible non-participants (non-responder or declined to participate)**

| **Thyroid Cancer** | | | | |
| --- | --- | --- | --- | --- |
| **Characteristic** | **Participated In Study** | | **Total *(n=278)*** | **p value** |
|  | **Yes *(n=102)*** | **No *(n=176)*** |  |  |
| **Sex (female, %)** | 64 (63%) | 126 (72%) | 190 (68%) | 0.13 |
| **Age (years) at diagnosis (mean, SD)** | 54.1 (14.9) | 49.8 (16.4) | 51.4 (16) | ***0.03*** |
| **Time (years) from diagnosis (mean, SD)** | 6.8 (4.6) | 6.4 (4.1) | 6.53 (4.3) | 0.46 |
| **Benign Thyroid Disease** | | | | |
| **Characteristic** | **Participated In Study** | | **Total *(n=418)*** | **p value** |
|  | **Yes *(n=104)*** | **No *(n=314)*** |  |  |
| **Sex (female, %)** | 83 (80%) | 273 (87%) | 356 (85%) | 0.08 |
| **Age (years) at diagnosis (mean, SD)** | 51.7 (13) | 48.3 (14.8) | 49.1 (14.4) | ***0.04*** |
| **Time (years) from diagnosis (mean, SD)** | 8.2 (5.2) | 7.9 (4.9) | 8.0 (4.9) | 0.66 |

**Supplementary Table 2:**

| **City of Hope-Thyroid Version** | **Thyroid Cancer Patients *(n=102)*** | **Benign Thyroidectomy *(n=104)*** | **Total *(n=206)*** | **p value** |  |
| --- | --- | --- | --- | --- | --- |
|  | *Mean (SD)* | *Mean (SD)* | *Mean (SD)* |  |  |
| **Physical subdomain questions** |  | | | |  |
| To what extent have the following been a problem during your illness and treatment: | | | | | |
| Fatigue | 5.8 (3.2) | 5.5 (2.9) | 5.6 (3) | 0.50 |  |
| Appetite changes | 7.7 (2.9) | 7.7 (2.7) | 7.7 (2.8) | 0.98 |  |
| Aches or pains | 7.1 (3) | 6.6 (3) | 6.9 (3) | 0.28 |  |
| Sleep changes | 6.4 (3.2) | 6 (3.1) | 6.2 (3.2) | 0.41 |  |
| Constipation | 8 (2.8) | 8.2 (2.6) | 8.1 (2.7) | 0.54 |  |
| Menstrual changes/fertility issues | 8.1 (3.3) | 8.2 (3.1) | 8.2 (3.2) | 0.86 |  |
| Weight gain | 6.6 (3.2) | 5.8 (3.3) | 6.2 (3.2) | 0.08 |  |
| Tolerance to cold or heat | 5.6 (3.3) | 5.9 (3.3) | 5.8 (3.3) | 0.50 |  |
| Dry skin or hair changes | 6.7 (3.2) | 6 (3.1) | 6.3 (3.2) | 0.10 |  |
| Voice changes | 7.5 (3.2) | 7.4 (3.1) | 7.4 (3.1) | 0.91 |  |
| Motor skills/coordination | 8.8 (2) | 8.3 (2.5) | 8.6 (2.3) | 0.10 |  |
| Swelling or fluid retention | 8.1 (2.7) | 7.9 (2.7) | 8 (2.7) | 0.60 |  |
| Rate your overall physical health | 6.5 (2) | 5.7 (1.9) | 6.1 (2) | ***0.005*** |  |
| *Total Physical Well-Being Score* | 7.1 (2) | 6.8 (1.8) | 7 (1.9) | 0.30 |  |
| **Psychological subdomain questions** | | | | | |
| How difficult is it for you to cope with your thyroid disease and treatment? | 8.2 (2.3) | 8.2 (2.5) | 8.2 (2.4) | 0.98 |  |
| How good is your QoL? | 7.2 (2.4) | 6.8 (2) | 7 (2.2) | 0.24 |  |
| How much happiness do you feel? | 7.1 (2.6) | 6.9 (2.6) | 7 (2.6) | 0.52 |  |
| Do you feel like you are in control of things in your life? | 7.2 (2.4) | 6.9 (2.5) | 7.1 (2.5) | 0.34 |  |
| How satisfying is your life? | 7.2 (2.6) | 7.1 (2.4) | 7.1 (2.5) | 0.84 |  |
| How is your present ability to concentrate or remember things? | 6.1 (2.5) | 5.4 (2.4) | 5.8 (2.4) | ***0.03*** |  |
| How useful do you feel? | 7.2 (2.5) | 6.7 (2.2) | 7 (2.4) | 0.15 |  |
| Has your thyroid illness or treatment caused changes in your appearance? | 7.3 (3.1) | 7.2 (3.1) | 7.2 (3.1) | 0.73 |  |
| Has your thyroid illness caused changes in the way you see yourself? | 7.7 (2.8) | 8.1 (2.8) | 7.9 (2.8) | 0.40 |  |
| How distressing were the following aspects of your illness and treatment: | | | | |  |
| Initial diagnosis | 3.8 (3.2) | 5.8 (3.4) | 4.8 (3.4) | ***<0.001*** |  |
| Surgeries | 4.4 (2.9) | 5.5 (3.3) | 5 (3.2) | ***0.02*** |  |
| Time since my treatment was completed | 7 (2.7) | 8.1 (2.3) | 7.5 (2.6) | ***0.006*** |  |
| Radioiodine ablation | 5.2 (3.3) | N/A | 5.3 (3.3) | 0.44 |  |
| Whole body scanning | 6.8 (3.4) | N/A | 7 (3.3) | 0.36 |  |
| Thyroglobulin testing | 8.2 (2.4) | N/A | 8.2 (2.4) | 0.90 |  |
| Withdrawal from thyroid hormone | 6.1 (3.8) | N/A | 6.2 (3.7) | 0.39 |  |
| How much anxiety do you have? | 6.3 (3) | 5.7 (2.9) | 6 (2.9) | 0.20 |  |
| How much depression do you have? | 7.1 (2.9) | 7 (3) | 7 (2.9) | 0.77 |  |
| To what extent are you fearful of: |  | | | |  |
| Future diagnostic tests | 7.4 (2.7) | 8.5 (2.4) | 7.9 (2.6) | ***0.003*** |  |
| A second cancer | 5.5 (3.2) | N/A | 5.5 (3.2) | 0.88 |  |
| Recurrence of your cancer | 6.1 (3.3) | N/A | 6.2 (3.3) | 0.42 |  |
| Spreading (metastasis) of your cancer | 5.9 (3.5) | N/A | 5.9 (3.5) | 0.52 |  |
| *Total Psychological Well-Being Score* | 6.6 (1.8) | 6.9 (1.6) | 6.8 (1.7) | 0.24 |  |
| **Social subdomain questions** | | | | | |
| How distressing has your thyroid illness been for your family? | 5.9 (3.1) | 7.6 (2.9) | 6.7 (3.1) | ***<0.001*** |  |
| Is the amount of support you receive from others sufficient to meet your needs? | 2.8 (3.1) | 3 (3.1) | 2.9 (3.1) | 0.54 |  |
| Is your continuing health care interfering with your personal relationships? | 8.4 (2.7) | 8.9 (2.3) | 8.6 (2.5) | 0.15 |  |
| Is your sexuality impacted by your illness? | 7.6 (3.4) | 7.7 (3.1) | 7.6 (3.3) | 0.78 |  |
| To what degree has your illness and treatment interfered with your employment: | | | | |  |
| Motivation to work | 7.5 (3) | 7.8 (2.8) | 7.6 (2.9) | 0.62 |  |
| Time away from work | 7.9 (2.9) | 8.4 (2.8) | 8.1 (2.9) | 0.24 |  |
| Productivity at work | 8 (2.8) | 7.9 (2.9) | 8 (2.8) | 0.72 |  |
| Quality of work | 8.4 (2.5) | 8.4 (2.4) | 8.4 (2.4) | 0.97 |  |
| To what degree has your illness and treatment interfered with your activities at home: | | | | |  |
| Driving a car | 9.5 (1.4) | 9.6 (1.3) | 9.5 (1.4) | 0.67 |  |
| Household chores | 8.4 (2.4) | 8.7 (2.3) | 8.5 (2.4) | 0.37 |  |
| Preparing meals | 8.7 (2.4) | 9 (1.9) | 8.8 (2.2) | 0.25 |  |
| Leisure activities | 8.3 (2.6) | 8.4 (2.5) | 8.3 (2.6) | 0.86 |  |
| How much isolation do you feel is caused by your illness and treatment? | 8.2 (2.9) | 9 (2.1) | 8.6 (2.6) | ***0.04*** |  |
| How much financial burden have you incurred as a result of your illness and treatment? | 7.1 (3.3) | 8 (2.7) | 7.6 (3.1) | ***0.04*** |  |
| *Total Social Well-Being Score* | 7.6 (1.8) | 8 (1.6) | 7.8 (1.7) | 0.09 |  |
| **Spiritual Subdomain Questions** | | | | | |
| How important to you is your participation in religious activities? | 2.8 (3.8) | 1.7 (3.1) | 2.3 (3.5) | ***0.03*** |  |
| How important to you are other spiritual activities such as meditation? | 2.1 (3.3) | 1.9 (3.3) | 2 (3.3) | 0.64 |  |
| How much has your spiritual life changes as a result of thyroid disease diagnosis? | 3.8 (3.1) | 2.9 (3.5) | 3.3 (2.9) | ***0.03*** |  |
| How much uncertainty do you feel about your future? | 6.9 (3) | 8.4 (2.5) | 7.6 (2.9) | ***0.0002*** |  |
| To what extent has your thyroid illness made positive changes in your life? | 2.6 (3.2) | 2.2 (3.1) | 2.4 (3.2) | 0.35 |  |
| Do you sense a purpose/mission for your life or a reason for being alive? | 5.9 (3.7) | 5.8 (3.6) | 5.8 (3.6) | 0.95 |  |
| How hopeful do you feel? | 7.1 (2.6) | 6.5 (3.3) | 6.8 (3) | 0.16 |  |
| *Total Spiritual Well-Being Score* | 4.5 (2) | 4.2 (1.7) | 4.3 (1.9) | 0.31 |  |
| **Total HRQoL Score** | **6.5 (1.4)** | **6.5 (1.2)** | **6.5 (1.3)** | **0.79** |  |
